# Reflux, dysphonia, and dysphagia symptoms in patients with diabetes and their association with diabetic complications

**DOI:** 10.55730/1300-0144.5372

**Published:** 2022-02-27

**Authors:** Hakan GÖLAÇ, Güzide ATALIK, Yusuf Kemal KEMALOĞLU, Metin YILMAZ

**Affiliations:** 1 Department of Speech and Language Therapy, Faculty of Health Sciences, Gazi University, Ankara, Turkey; 2 Department of Otolaryngology, Faculty of Medicine, Gazi University, Ankara, Turkey

**Keywords:** Diabetic complications, reflux, swallowing disorders, voice disorders

## Abstract

**Background/aim:**

Diabetes mellitus (DM) could influence various organs, especially the eyes, kidneys, nerves, heart, and blood vessels, and finally results in many irreversible disease-related complications. In this paper, the association between reflux, swallowing, and voice symptoms in patients with DM and the possible effect of diabetic complications on these symptoms were investigated.

**Materials and methods:**

A total of 179 patients with diabetes were included to the study. Three self-reported questionnaires; Reflux Symptom Index (RSI), Eating Assessment Tool-10 (EAT-10), and Voice Handicap Index-10 (VHI-10) were administrated to the patients and, their association with DM-related neuropathy and nephropathy were examined.

**Results:**

The scores of each questionnaire were significantly correlated with each other (p < 0.001). There was not any statistically significant association between the score of T-RSI and the diabetic complications (p = 0.077), while a statistically significant association was found between the T-EAT-10 score and neuropathy (p < 0.001). Neither neuropathy nor nephropathy alone had an association with the T-VHI-10 score. However, the presence of nephropathy and neuropathy together was found to be associated with the T-VHI-10 score (p = 0.027).

**Conclusion:**

It is possible to conclude that gastrointestinal symptoms such as reflux, dysphonia, and dysphagia are associated with each other and they may possibly be related to the microvascular complications of DM. The clinicians should be aware of the possible reflux, voice, and swallowing complaints and also inquire about the presence of neuropathy and nephropathy in the diabetic population.

## 1. Introduction

Diabetes mellitus (DM) is a metabolic disorder characterized by chronic hyperglycemia and its incidence and prevalence are increasing all over the world. The prevalence was predicted to be 9.3% (463 million people) in 2019, 10.2% (578 million) in 2030 and 10.9% (700 million) in 2045 by The International Diabetes Federation (IDF) [1]. Currently, DM is one of the most common chronic diseases and a growing public health concern globally due to its high rate of morbidity and mortality [2,3].

Diabetic complications which are classified as microvascular or macrovascular are a major cause of morbidity and mortality. While microvascular complications consist of neuropathy, nephropathy, and retinopathy; macrovascular complications include peripheral vascular disease, cerebrovascular disease, and cardiovascular disease [4]. DM-related complications like diabetic neuropathy are considered to be the primary reason for gastrointestinal (GI) tract symptoms [5]. It is well established that GI symptoms such as; reflux, dysphagia, vomiting, nausea, heartburn, abdominal pain or discomfort, diarrhea or constipation, and bloating are more common among patients with diabetes than the general population. [6–8]. Particularly, the prevalence of gastroesophageal reflux disease (GERD) is relatively high in patients with DM and it is reported to be associated with the microvascular complications of DM [9,10]. On the other hand, in a study by Hamdan et al. [11], laryngopharyngeal signs and symptoms such as; throat clearing, globus, vocal fatigue, and dysphonia were found to be prevalent in patients with GERD. Also, a number of studies have addressed that laryngopharyngeal symptoms may be predictors of gastroesophageal diseases and GERD and they are associated with extraesophageal reflux disease, termed laryngopharyngeal reflux (LPR) [11,12]. Besides, in the diabetic population, patients were reported to be more likely to have LPR and phonatory symptoms compared with controls [13,14]. Briefly, diabetic complications may be an important etiological cofactor in the development of GI tract symptoms, particularly in reflux, dysphagia, and dysphonia.

Self-reported questionnaires are used to assess the impacts of health problems on the quality of life of patients. Reliability, convenience, rapid administration time, noninvasiveness, and low-cost are the primary reasons for using questionnaires in clinical settings [15]. In the present study, we used three validated self-reported questionnaires namely, The Turkish Reflux Symptom Index (T-RSI), The Turkish Eating Assessment Tool-10 (T-EAT-10), The Turkish Voice Handicap Index-10 (T-VHI-10) to collect data about the patients’ reflux, swallowing, and voice complaints, respectively. To the best of our knowledge, there is no study investigating the association between reflux, dysphagia, and dysphonia in DM by using the above-mentioned questionnaires.

Specifically, we hypothesize that the score of the questionnaires are correlated with each other and diabetic complications are associated with abnormal questionnaire scores. The primary aim of the current study is to investigate the association between reflux, swallowing, and voice complaints in patients with diabetes. We further aimed to identify the possible effect of diabetic complications, namely, neuropathy and nephropathy, on reflux, dysphonia, and dysphagia symptoms in the target population.

## 2. Materials and methods

### 2.1. Participants

An observational cross-sectional research model was used to collect data from the adult patients with DM, who were followed up at Gazi University Diabetes-Obesity Outpatient Clinic. A total of 179 subjects (76 males and 103 females) with a mean age of 55.16 ± 10.21 and a range of 24–81 years were recruited to the present study between January 2019 and May 2019. The subjects with any additional diagnosis that could affect swallowing and voice functions, those with a prior history of head and neck or GI tract surgery, and those who are ineligible to fill in the questionnaires were excluded from the study. However, for the patients who were able to understand and answer the questions, but unable to fill in the questionnaires on their own, each item of the questionnaires was read to them by the researchers. Ethical approval was gained from the Gazi University Research Ethics Committee. Prior to enrollment, a written informed consent was required from all subjects.

### 2.2. Data collection

The following characteristics for each patient were noted: age, sex, body mass index (BMI) based on self-reported height and weight, duration of DM, the level of HgA1c, and presence of DM-related neuropathy and nephropathy. In our hospital, a clinically based diagnosis of neuropathy is routinely made by an endocrine physician at the time of the clinical visits. Positive sensory findings such as numbness, tingling, burning, and pain in the limbs are considered and noted for the presence of neuropathy. For the diagnosis of nephropathy, proteinuria and microalbuminuria levels and renal echography are regularly made and recorded in patients’ charts. In the current study, the presence of both neuropathy and nephropathy were acquired from the electronic medical record of each participant. Next, the patients were required to complete the following three questionnaires, namely: Reflux Symptom Index (RSI) [16], Eating Assessment Tool-10 (EAT-10) [17], and Voice Handicap Index-10 (VHI-10) [18] under the supervision of the first two authors. Validation and cultural adaptation of the Turkish version of the above-mentioned questionnaires are available in the current literature [19–21].

### 2.3. The questionnaires

The Turkish Reflux Symptom Index (T-RSI) [19] is a reliable and valid self-administered clinical tool for the assessment of LPR. It consists of nine items and each item score ranges from 0 (no problem) to 5 (severe problem). While a maximum score of 45 indicates the most severe symptoms, a score greater than 13 is considered to be abnormal.

The Turkish Eating Assessment Tool-10 (T-EAT-10) [20] is a practical and easily self-administered, 10-item swallowing screening tool. Each item has a 5-point scale ranging from 0 (no difficulty) to 4 (severe difficulty) and a score of 3 or higher is considered to be an abnormal swallowing function.

The Turkish Voice Handicap Index-10 (T-VHI-10) [21], was used for self-evaluation of voice problems. The questionnaire consists of 10 voice-related items, with five possible answers: 0–never; 1–almost never; 2–sometimes; 3–almost always; and 4–always. The higher score means the higher level of dysphonia and the subjects with a cutoff score of > 11 are considered to have voice-related problems [22].

### 2.4. Statistical analysis

The normality of data distribution was examined using the visual (histogram and probability graphs) and analytical methods (Kolmogorov-Smirnov/Shapiro-Wilk tests). While all categorical variables were presented as percentages, all normally distributed variables were presented as mean and standard deviations (SD) and all skewed variables were presented as median (minimum-maximum value). Spearman correlation was used to assess the level of correlation between scores of the questionnaires and other clinical variables. A chi-square test was applied to investigate the association between diabetic complications and scores of the questionnaires. All data analyses were performed using Statistical Package for Social Sciences (SPSS) version 22.0 (SPSS Inc. Chicago, USA), with the level of significance at p < 0.05.

## 3. Results

In all 179 subjects participated to the study, the median duration of DM was 8 years (range: 1 to 41 years) and the median value of HgA1c was 7.1 (range: 4.9 to 13.8). Out of the total 179 patients, 44 (24.6%) and 26 (14.5%) had diabetic neuropathy and diabetic nephropathy, respectively. The detailed patients’ characteristics and clinical findings are given in Table 1.

**Figure 1:**
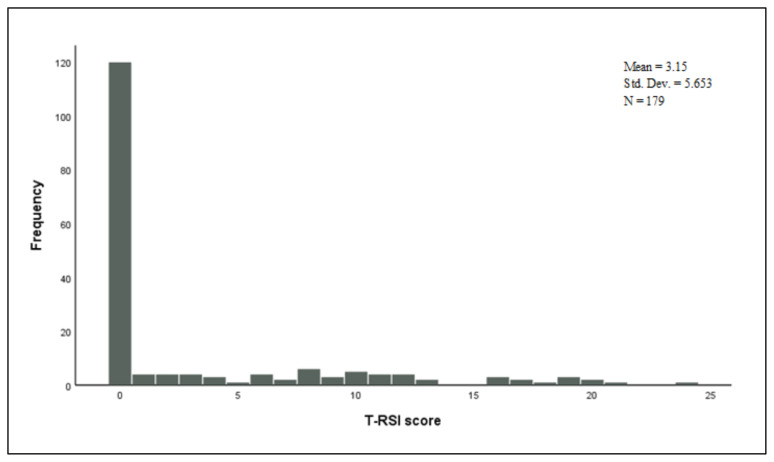
Distribution of the Turkish-Reflux Symptom Index scores.

**Figure 2:**
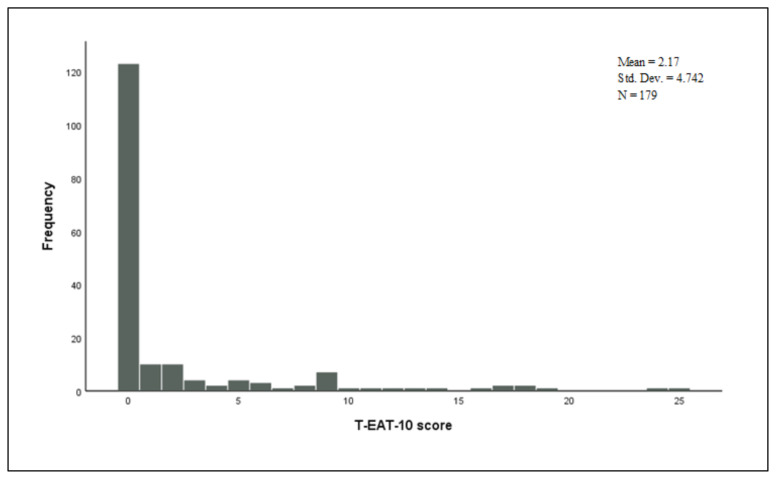
Distribution of the Turkish-Eating Assessment Tool-10 scores.

**Figure 3:**
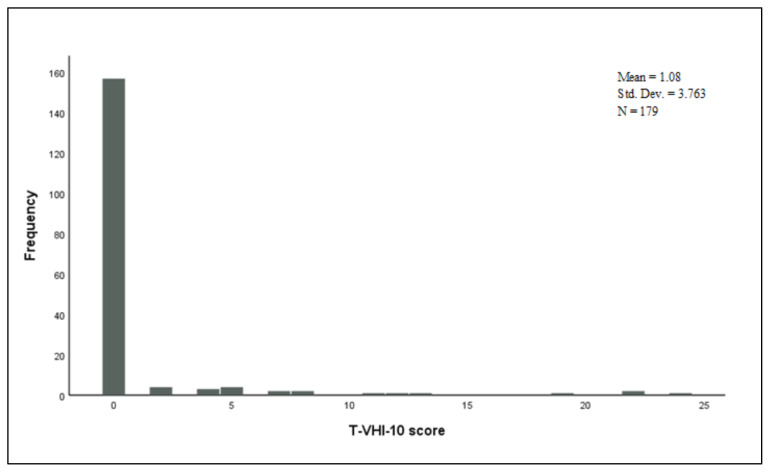
Distribution of the Turkish-Voice Handicap Index-10 scores.

Thirteen patients (7.3%) had an abnormal T-RSI score (a score of >13) and 36 (20.1%) of the subjects had an abnormal T-EAT-10 score (a score of ≥3). The number of the participants with an abnormal score of >11 from the T-VHI-10 questionnaire was 7 (3.9%) (See Table 2 for more details). The distributions of the scores obtained from each questionnaire are illustrated in Figures 1, 2, and 3.

The Spearman analyses presented that duration of DM and age (r: 0.320) and HgA1c (r: 0.212) were correlated to each other. Among the questionnaires, only T-VHI-10 scores were correlated with age (r: −0.106) and duration of the DM (r: 0.002). However, no correlation was found between the HgA1c level and all three questionnaires. It was seen that scores of each questionnaire were significantly correlated with each other (p < 0.001) (Table 3).

As it is seen in Table 4, there was not any statistically significant association between the score of T-RSI and the diabetic complications (p = 0.077), while a statistically significant association was found between the T-EAT-10 score and neuropathy (p < 0.001). Among the diabetic complications, neither neuropathy nor nephropathy alone had an association with the T-VHI-10 score. However, the presence of nephropathy and neuropathy together was found to be associated with the T-VHI-10 score (p = 0.027).

## 4. Discussion

Most of the patients with diabetes have insulin defects in insulin secretion, insulin action, or both [23]. These metabolic changes if not controlled lead to mortalities and morbidities in various organs, especially in the eyes, kidneys, nerves, heart, and blood vessels [23,24]. While the microvascular complications start to develop with the onset of hyperglycemia, the macrovascular complications develop at the onset of insulin resistance.

Neuropathy is one of the most common microvascular complications of diabetes. In clinic-based studies, the prevalence of neuropathy in people with DM has ranged from 16% to 26% [25–27]. As it is known, nephropathy is another DM-related complication that is commonly encountered in patients with diabetes and its incidence was stated to be in the range of 6.7% to 42.5% [28,29]. In line with the literature, the present study revealed that 24.6% and 14.5% of the total 179 participants had neuropathy and nephropathy respectively.

To date, a growing number of studies have been conducted to investigate the voice and swallowing functions in the diabetic population [14,30–33]. However, to the best of our knowledge, this is the first study to attempt to understand the possible effect of neuropathy and nephropathy, on reflux, dysphonia, and dysphagia symptoms in DM patients. On the other hand, there is not any report in the literature about the association between reflux, swallowing, and voice complaints in patients with diabetes.

Hamdan et al. [14] studied the prevalence of phonatory symptoms in patients with type 2 DM and reported that diabetic patients are more likely to have vocal straining and hoarseness compared to controls. In another research by Hamdan et al. [31], they compared the vocal characteristics of patients with type 2 DM with healthy controls. Despite no statistically significant difference in any of the perceptual evaluation parameters namely; grade, roughness, breathiness, asthenia, and straining (GRBAS) between the groups, the mean scores were all higher in the diseased group except for roughness. In both studies, the presence of neuropathy was stated to affect vocal health. In the current study, the presence of nephropathy and neuropathy together was found to be associated with the T-VHI-10 score (p = 0.027). Accordingly, the T-VHI-10 scores of individuals who have nephropathy and neuropathy together were high.

When the effect of neuropathy and nephropathy on swallowing function was considered, there was a statistically significant association between the T-EAT-10 score and neuropathy but not for nephropathy. In a previous study that was completed with 121 participants with Type 2 DM, Gölaç et al. [30] reported that T-EAT-10 score was significantly associated with neuropathy and reflux complaint which was similar to our findings.

Recent evidence suggests that the prevalence of GERD is relatively high in patients with DM and it is associated with LPR [11,12]. Also, in several studies, reflux is reported to be associated with DM-related microvascular complications [9,10]. However, our findings did not correlate well with the previous literature that no statistically significant association was found between the diabetic complications and T-RSI score. The low number of participants was considered to be the probable reason and it is thought that an association could be found in a study with more participants. Additionally, the proportion of patients having reflux symptoms was 7.3%, which seems to be less than the reported prevalence (25%–41%) in the literature [34,35]. To date, many studies have been conducted to investigate the effectiveness of diet modification and lifestyle change on the improvement of symptoms, signs, and voice quality in reflux disease [36–39]. It is generally believed that modification in dietary contents of the patients appears to have beneficial effects on the symptoms and findings of reflux and yields rapid and substantial results in treating reflux-related complaints. In the current study, there is no data regarding the diet contents of the patients which may likely influence the reflux-related signs and symptoms. Therefore, the reason for the low prevalence of reported reflux in our patient group might be due to the diet contents of the participants.

It has been stated in many studies that the presence of reflux alone may cause voice and/or swallowing complaints [40–42]. In the present study, the scores of each questionnaire were significantly correlated with each other. The probable reason for this correlation is that all the items of the questionnaires reveal the findings related to GI tract symptoms and/or discomforts. In the validity and reliability study of the EAT-10, the mean score of the questionnaire was reported to be high for those with reflux disease and voice disorder. Also, no significant difference was found in the EAT-10 score of those with a voice disorder and those with reflux disease. Therefore, patients with diabetes, particularly those who have reflux should be considered about the probable dysphonia and dysphagia.

The present study has several limitations that warrant consideration. First, the presence of neuropathy and nephropathy was determined with the clinical symptoms and signs. It is of importance to use objective methods in the diagnosis of these microvascular complications that can give more accurate results. Second, this study was designed as an observational cross-sectional research model and a relatively small subgroup of patients who had reflux and voice complaints were included in data analyses. Thus, the causal association between variables is not firmly established in this study. Further studies with a control group and with a relatively larger sample size are necessary to determine whether these findings can be generalized to the diabetic population.

From the research that has been conducted, it is possible to conclude that GI symptoms such as reflux, dysphonia, and dysphagia are associated with each other in patients with DM. Also, these symptoms may possibly be related to the microvascular complications of diabetes, such as neuropathy and nephropathy. Therefore, in the diabetic population, the clinicians should be aware of the possible reflux, voice, and swallowing complaints and also inquire about the presence of neuropathy and nephropathy. Because the self-administrated questionnaires are easy to use and cost-effective in clinical practice, they can be used as a screening tool to detect reflux, voice, and swallowing-related problems of patients with diabetes.

## Figures and Tables

**Table 1:** Patients’ characteristics and clinical findings.

Variables	DM N = 179

**Age, years** (mean ± sd)	55.16 ± 10.21

**Gender, n (%)** *	
Female	103 (57.5)
Male	76 (42.5)

**BMI, kg/m^2^** median (range)	29.7 (19.5–53.7)

**Duration of diabetes, years** median (range)	8 (1–40)

**HgA1c, %** median (range)	7.1 (4.9–13.8)

**Nephropathy + neuropathy, n (%)**	14 (7.8)

**Nephropathy, n (%)**	12 (6.7)

**Neuropathy, n (%)**	30 (16.8)

**No complications, n (%)**	123 (68.7)

DM, Diabetes mellitus;

* , Column percentage;

BMI, Body mass index; HgA1c, Hemoglobin A1C

**Table 2:** Scores of the questionnaires.

Variables	DM n = 179

**T-RSI score**, median (range)	0 (0–24)

**T-RSI score, n (%)** *	
Normal (score ≤ 13)	166 (92.7)
Abnormal (score > 13)	13 (7.3)

**T-Eat-10 score**, median (range)	0 (0–25)

**T-Eat-10 score, n (%)**	
Normal (score < 3)	143 (79.9)
Abnormal (score ≥ 3)	36 (20.1)

**T-VHI-10 score**, median (range)	0 (0–24)

**T-VHI-10 score, n (%)**	
Normal (score ≤ 11)	172 (96.1)
Abnormal (score > 11)	7 (3.9)

* , Column percentage;

T-Eat-10, Turkish Eating Assessment Tool-10; T-RSI, Turkish Reflux Symptom Index; T-VHI-10, Turkish Voice Handicap Index-10

**Table 3:** Spearman correlation between scores of the questionnaires and other clinical variables.

	Age		Duration of DM		HgA1c		T-RSI score		T-EAT-10 score		T-VHI-10 score	
	r	p-value		p-value	r	p-value	r	p-value	r	p-value	r	p-value
**Age**	-	-										
**Duration of DM**	0.320	< 0.001	-	-								
**HgA1c**		NS	0.212	0.004	-	-						
**T-RSI score**		NS		NS		NS	-	-				
**T-EAT-10 score**		NS		NS		NS	0.371	< 0.001	-	-		
**T-VHI-10 score**	−0.106	0.033	−0.225	0.002		NS	0.378	< 0.001	0.398	< 0.001	-	-

NS, Not significant; DM, Diabetes mellitus; HgA1c, Hemoglobin A1C; T-Eat-10, Turkish Eating Assessment Tool-10; T-RSI, Turkish Reflux Symptom Index; T-VHI-10, Turkish Voice Handicap Index-10

**Table 4:** Association between the diabetic complications and scores of the questionnaires.

	Nephropathy + neuropathy (n = 14)	Nephropathy (n = 12)	Neuropathy (n = 30)	No complications (n = 123)	p-value

**T-RSI score, n (%)** *					
Normal (score ≤ 13)	12 (7.2)a	11 (6.6)a	25 (15.1)a	118 (71.1)a	0.077
Abnormal (score > 13)	2 (15.3)a	1 (7.7)a	5 (38.5)a	5 (38.5)a	

**T-Eat-10 score, n (%)**					
Normal (score < 3)	8 (5.6)a	10 (7.0)a,b	13 (9.1)a	112 (78.3)b	< 0.001
Abnormal (score ≥ 3)	6 (16.7)a	2 (5.6)a,b	17 (47.2)a	11 (30.6)b	

**T-VHI-10 score, n (%)**					
Normal (score ≤ 11)	12 (7.0)^a^	12 (7.0) a,b	27 (15.7) a,b	121 (70.3)b	0.027
Abnormal (score > 11)	2 (28.6)^a^	0 (0) a,b	3 (42.8) a,b	2 (28.6)b	

* , Row percentage;

T-Eat-10, Turkish Eating Assessment Tool-10; T-RSI, Turkish Reflux Symptom Index; T-VHI-10, Turkish Voice Handicap Index-10

Each superscript letter denotes a subset of categories whose column proportions do not differ significantly from each other at the 0.05 level (categories that do not share a common superscript letter are significantly different from each other).
